# The role of nitric oxide signaling in food intake; insights from the inner mitochondrial membrane peptidase 2 mutant mice^☆^

**DOI:** 10.1016/j.redox.2013.10.003

**Published:** 2013-10-24

**Authors:** Changjie Han, Qingguo Zhao, Baisong Lu

**Affiliations:** a Wake Forest University Health Sciences, Institute for Regenerative Medicine, Winston-Salem, NC 27157, United States; b Department of Human Anatomy and Embryology, The Second Military Medical University, Shanghai 200433, China

**Keywords:** *Immp*2*l*, IMP2 inner mitochondrial membrane peptidase-like, cGMP, cyclic guanosine monophosphate, ROS, reactive oxygen species, UCP2, uncoupling protein 2, NPY, neuropeptide Y, AgRP, agouti related protein, POMC, pro-opiomelanocortin, NO, nitric oxide, NOS, nitric oxide synthase, CART, cocaine- and amphetamine-regulated transcript, CYC1, cytochrome c1, GPD2, mitochondrial glycerol phosphate dehydrogenase, ADSC, adipose-derived stromal cells, AMPK, AMP-activated protein kinase., *Immp*2*l*, Mutant mice, Food intake, Superoxide, Nitric oxide, Energy expenditure

## Abstract

Reactive oxygen species have been implicated in feeding control through involvement in brain lipid sensing, and regulating NPY/AgRP and pro-opiomelanocortin (POMC) neurons, although the underlying mechanisms are unclear. Nitric oxide is a signaling molecule in neurons and it stimulates feeding in many species. Whether reactive oxygen species affect feeding through interaction with nitric oxide is unclear. We previously reported that *Immp*2*l* mutation in mice causes excessive mitochondrial superoxide generation, which causes infertility and early signs of aging. In our present study, reduced food intake in mutant mice resulted in significantly reduced body weight and fat composition while energy expenditure remained unchanged. Lysate from mutant brain showed a significant decrease in cGMP levels, suggesting insufficient nitric oxide signaling. Thus, our data suggests that reactive oxygen species may regulate food intake through modulating the bioavailability of nitric oxide.

## Highlights

• Mature adult *Immp*2*l* mutant mice have reduced body weight and fat composition.
• Reduced body weight and fat composition is caused by reduced food intake.
• Energy expenditure is not affected in mutant mice.
• Brain cGMP level is lower in mutant mice.

## Graphical abstract

**Figure:**
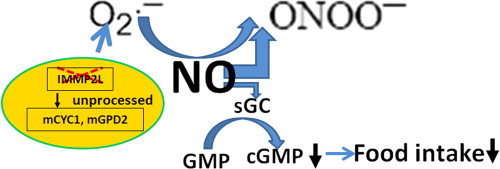

## Introduction

Reactive oxygen species (ROS) are implicated in feeding regulation through various mechanisms. For example, ROS are involved in brain lipid sensing, which regulates food intake [1]. Additionally, regulation of free radical generation in the mitochondria by UCP2 mediates ghrelin's action on NPY/AgRP neurons [2]; suppression of ROS promotes feeding by diminishing the activation of pro-opiomelanocortin (POMC) neurons and enhancing the activity of NPY/AgRP neurons, whereas expression of ROS activates POMC neurons and reduces feeding [3]. Whether alternate mechanisms are involved in ROS regulation of feeding requires further investigation.

Nitric oxide (NO), a signaling messenger, is involved in feeding control in multiple species, including chicken [4,5], mice [6–9] and rats [10,11]; moreover, NO is a central component in neuropeptide regulation of appetite [12]. Blockade of NO synthesis reduces adiposity in high fat-induced obese mice [13] and obese Zucker rats [14]. Three nitric oxide synthases (NOS), the constitutive neuronal NOS (nNOS), endothelial NOS (eNOS), and the inducible NOS (iNOS), are responsible for the synthesis of NO. Leptin, a hormone produced by adipocytes, inhibits brain NO synthesis and decreases food intake [15,16]. Guanylate cyclase, the principal NO receptor, synthesizes cyclic guanosine monophosphate (cGMP) upon activation by NO.

The hypothalamus is a critical part of the brain that contains nuclei, such as the arcuate nucleus (ARC), the ventromedial nucleus (VMN) and the dorsomedial nucleus (DMN), which contain multiple neurons that receive signals from insulin and leptin to regulate food intake [17–19]. In the ARC, neuropeptide Y (NPY) and Agouti-related peptide (AgRP), which are co-expressed by NPY/AgRP neurons, stimulate food intake [20–24]. Gene products from pro-opiomelanocortin (POMC) and cocaine- and amphetamine-regulated transcript (CART), which are co-expressed by POMC neurons, suppress food intake [25,26]; therefore, ROS may be acting through NPY/AgRP and POMC neurons [2,3]. Pharmacological activation of AMP-activated protein kinase (AMPK) in the hypothalamus will cause increased food intake [27], NO production, and neuronal activity [28]. AMPK is activated via phosphorylation of threonine 172 of AMPKα subunit [29].

Superoxide and NO have opposing effects on blood vessel contraction [30]. The reaction of superoxide with NO yields peroxynitrite at a speed several times faster than its own dismutation by superoxide dismutases [31–33]. Recently, we generated the *Immp*2*l* mutant mouse model through random transgenic insertional mutagenesis [34,35]. IMMP2L is a mitochondrial inner membrane peptidase, which cleaves the space-sorting signal peptide sequences from cytochrome c1 (CYC1) and mitochondrial glycerol phosphate dehydrogenase (GPD2). Accordingly, the signal peptide sequences of CYC1 and GPD2 are not cleaved in *Immp*2*l* mutant mice. The mitochondria from mutant mice generate excessive superoxide ions, but they show no obvious deficiencies in ATP generation and membrane potential maintenance [35]. Mutant mice exhibit erectile dysfunction [35], defective oogenesis [35] and bladder dysfunction [36], which may result from inactivation of NO by superoxide. The mutant mice also develop age-dependent spermatogenic impairment [35,37], accelerated aging [38], and increased ischemic brain damage [39] due to increased oxidative stress.

While studying the effects of mitochondrial superoxide over-generation on aging, we noted that mutant mice have significantly reduced body weight and fat composition compared to control mice after the age of 5 months. Data from our present study suggest that decreased food intake may account for reduced body weight and adiposity in mutant mice; furthermore, our data suggest that the mechanism of food intake decrease may be the decreased bioavailability of NO in the brain of mutant mice.

## Material and methods

### Animals

The generation of *Immp*2*l*^*Tg(Tyr)*979*Ove*^/*Immp*2*l*^*Tg(Tyr)*979*Ove*^ mutant mice has been described previously [35], and the mice have been maintained on an FVB/N background. *Lep*^*ob/ob*^ mice have been described previously [40], and animals were purchased from The Jackson Laboratories. The mice were backcrossed to FVB/N background for five generations before crossing with *Immp*2*l* heterozygous mice. *Immp*2*l* and *Leptin* double mutant mice were generated by mating double heterozygous males and females. Mice were housed in the pathogen-free animal facility of Wake Forest University Health Sciences. Experiments were conducted in accordance with the National Research Council publication Guide for Care and Use of Laboratory Animals, and approved by the Institutional Animal Care and Use Committee of Wake Forest University Health Sciences. Mice were kept in microisolator cages with 12-h light/dark cycles and were fed ad libitum on a chow diet (Prolab, RMH3000). Genotypes of the mice were determined by coat color [35].

### Food consumption

Food consumption was determined for mice that were individually caged in cages with lifted wire bottoms, as described previously [41]. Food spilled on the bottom of the cages was recovered and subtracted from food intake. Unless otherwise stated, the number of animals per group was equal to or greater than five. Food intake was assayed prior to noticeable differences in body weight between control and mutant mice. Food intake was normalized to body mass and analyzed by Student's *t*-test, and *p* values <0.05 were regarded as statistically significant.

To compare fasting induced food intake, mice were individually housed in cages with lifted wire bottoms, fasted for 15 h (from 6:00 pm to 9:00 am) or 16 h (from 10:00 pm to 2:00 pm), and then provided with food. Food intake during the first 4 h was recorded and compared by Student's *t*-test.

### Blood biochemical assays

Sera were collected from mice following 6 h fasting. Free fatty acids were assayed with kits from Cayman Chemical (Ann Arbor, MI). Serum leptin, insulin, cholesterol and triglyceride were assayed as described previously [41].

### Determination of cGMP and nitrate/nitrite concentrations

To avoid cGMP degradation, mice were euthanized by cervical dislocation and brain removed within 2–3 min after death. The hypothalami were dissected and snap frozen in liquid nitrogen, and stored at −80 °C prior to measuring hypothalamic cGMP concentrations. The hypothalamic tissues were lysed in 5% trichloroacetic acid (10 ml solution per 1 g tissue) and extracted with water-saturated ether. A Monoclonal Anti-cGMP EIA Kit without Acetylation from Neweast Biosciences Inc. (Cat# 80103) was used to compare cGMP according to manufacturer's protocol. Total nitrate and nitrite contents in the trichloroacetic acid extracts were assayed with a kit from Cayman Chemical.

### Magnetic resonance imaging (MRI) analysis of fat composition

Fat composition was determined by MRI using the Bruker Biospin 7 T microMRI scanner housed in the Center for Biomolecular Imaging, Wake Forest University Health Sciences as described previously [41].

### Metabolic studies

Indirect calorimetry and locomotor activity were measured using the Oxymax CLAMS system (Columbus Instruments). Animals were tested at 4 months of age and prior to noticeable differences in body weight and fat composition. Oxygen consumption rate (VO_2_) and CO_2_ production rate (VCO_2_) in individual mice were measured using metabolic chambers, and the respiratory exchange ratio (RER) was calculated to reflect energy expenditure. A photobeam-based activity monitoring system was used to detect and record ambulatory movements. The temperature of the cabinet was set at 26 °C. Energy expenditure (kJ/h) was calculated using the formula: VO_2_×[3.815+(1.232×RER)]×4.1868) [42] and was normalized to body weight since control and mutant mice showed similar body weight at the time of assay. All parameters were measured continuously and simultaneously for 72 h after 36 h of adaptation in singly housed mice. Three-day averages for each mouse were used for analysis.

### Quantitative RT-PCR analysis of gene expression

Total RNA was extracted from hypothalamic tissues with Turbo DNase (Ambion) to eliminate DNA contamination, and reverse transcribed as described previously [43]. Primers for *Npy*, *Agrp* and *Pomc* have been described in our recent publication [41]. Other primers used include: pmchF (agcggtttcatgaacgatg) and pmchR (tcagacttgccaacatggtc) for *Pro-melanin-concentrating hormone*, OrexinF (gggtatttggaccactgcac) and OrexinR (ttcgtagagacggcaggaac) for *Orexin* gene, sGCF (ctgctggtgatccgcaattat) and sGCR (gatggtatcatagccagactcct) for *Soluble guanylyl cyclase*, eNOSF (ccttccgctaccagccaga) and eNOSR (cagagatcttcactgcattggcta) for *eNos*, nNOSF (tcctaaatccagccgatcga) and nNOSR (tcatggttgccagggaagac) for *nNos*, and iNOSF (agagagatccgatttagagtcttggt) and iNOSR (tgacccgtgaagccatgac) for *iNos*. Equal amounts of RNA from five mice per group were mixed for RT-PCR analysis. Mean±SEM was obtained from three to four independent PCR analyses, with each PCR analysis including triplicate wells.

### SDS–PAGE and Western blotting analyses

Hypothalamic tissue were homogenized in RIPA buffer containing protease inhibitors (0.5 mM PMSF and 1× Complete Protease Inhibitor Cocktail, Roche) and phosphatase inhibitors (50 mM NaF, 1.5 mM Na_3_VO_3_). Anti-β-actin antibody was from Sigma; antibody to phosphorylate AMPK was from Millipore. Horseradish peroxidase (HRP)-conjugated secondary antibodies were purchased from Pierce. Chemiluminescent reagents from Pierce were used to visualize the protein signals under the LAS-3000 system from Fujifilm. ImageJ software was used to quantify the expression of individual protein bands and normalized to β-actin. Student's *t*-test was used to compare protein expression between control and mutant mice.

### Adipose-derived stromal cell (ADSC) isolation and culture

Gonadal white adipose tissues were weighed, minced, and digested with 0.1% collagenase I for 1 h at 37 °C. The digested tissues were centrifuged at 3000 rpm for 10 min to separate adipose-derived stromal cells (ADSCs) from mature fat cells. The isolated ADSCs were cultured in MEM-α medium (Invitrogen) supplemented with 10% FBS, 5 ng/ml FGF2, and antibiotics. Freshly isolated ADSCs (1/50 cells from the mouse with the smallest tissue, the remaining mice were normalized by adipose tissue mass) were plated in 6-well dishes. After 10 days of culture, the cells were fixed with methanol for 5 min at room temperature and stained for 30 min with Giemsa staining. For proliferation assay, the cells were plated at a density of 5×10^4^ cells/well in 6-well plates. After reaching confluence, the cells were trypsinized and counted and 5×10^4^ cells were re-plated for expansion.

## Results

### Immp2l mutant mice have reduced body weight and fat deposition

**Fig. 1:**
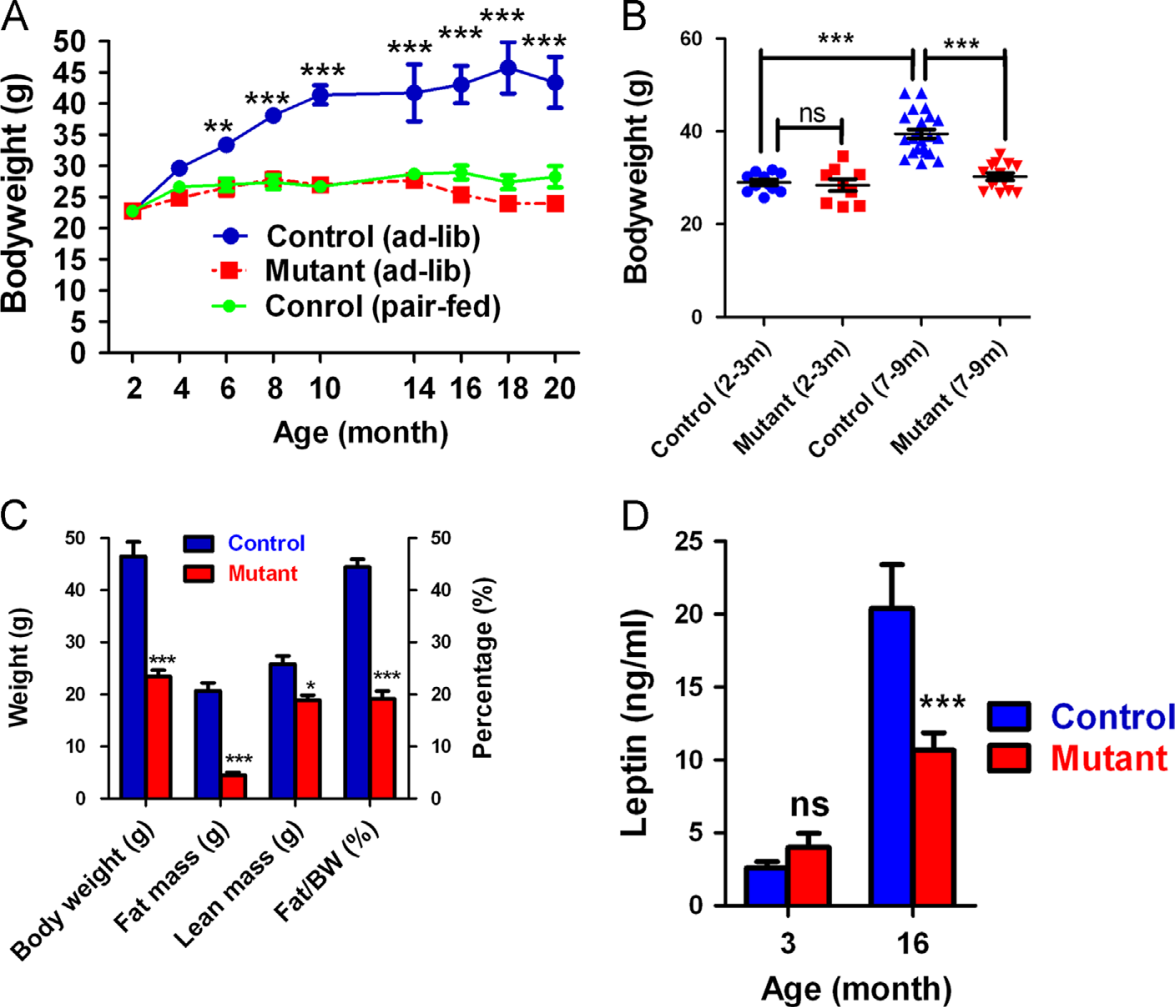
Reduced body weight and adiposity of *Immp*2*l* mutant mice. (A) Body weight of female mice at different ages. Ad-lib indicates fed ad libitum. (B) Body weight of male mice. (C) Body weight and fat composition of 16-month-old female mice. Total fat composition was determined by MRI. Means±SEM. for *n*≥5 animals per group are represented. *, ** and ***, *p*<0.05, *p*<0.01 and *p*<0.0001 respectively by Bonferroni post-tests ((A), (C) and (D)) and Tukey post-tests (B) following ANOVA.

Both male and female adult *Immp*2*l* mutant mice gained little body weight from 3 months to 20 months, while control mice gained significant weight during this time (Fig. 1A, females, and Fig. 1B, males). Since mutant mice did not show age-associated symptoms until 16 months [38], failure to gain weight cannot be explained by accelerated aging. Comparing fat composition between control and mutant female mice at 15 months of age revealed that mutant mice had significantly reduced fat mass and fat composition than control mice, and reduced fat mass accounted for a major part of the body weight difference (Fig. 1C).

Blood lipids, insulin and leptin concentrations were compared between control and mutant mice (Table 1). These parameters were similar between control and mutant mice at 3 months of age when body weights were similar. Control mice, however, had significantly higher leptin, insulin and triglyceride levels than mutant mice after 12 months of age, and they showed higher adiposity than mutant mice. Free fatty acids and cholesterol levels were similar between control and mutant mice regardless of age.

### Mutant mice have reduced food intake

Reduced adiposity in mutants could be caused by reduced food intake, increased energy expenditure, or both. Previously, we observed no difference in food intake/body weight ratios between 6 and 7-month-old control and mutant mice [38]. At this age, control and mutant mice showed significant difference in body weight and fat composition; thus, food intakes were compared at 3 months of age and their body weight showed no difference. The absolute daily food intake and food/body weight ratio were both reduced by 30% in mutant mice (Table 2). Although mutant mice had an earlier recorded reduced food intake, their body weight was not different from control mice until after 5 months. This may be due to low adiposity in young mice, including control animals, and differences are detectable as the animal's age and adipose tissue constitutes a larger percentage of total body weight.

As observed in female mice, differences in body weight of older male mice were a result of variable accumulation of fat (data not shown). Food intake was compared in 13-month-old male mice. Consistent with data in Fig. 1B, control males had significantly higher body weight than mutant males at the same age (Table 2). Food intake of control male mice was larger than that of mutant males, but the difference did not achieve statistical significance. An increased sample size would be necessary to observe the difference.

**Fig. 2:**
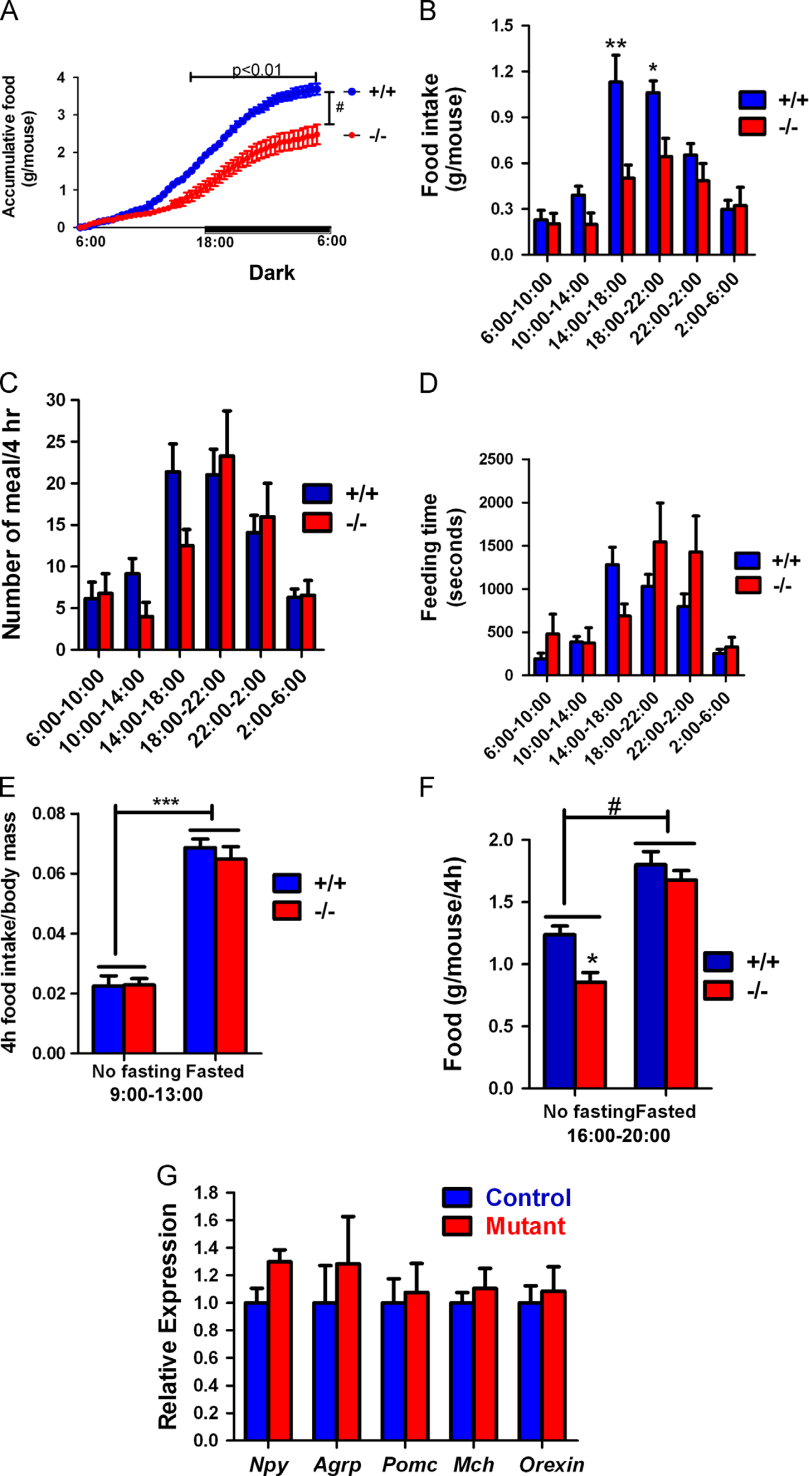
Reduced food intake in mutant mice. (A) Accumulative food intake during 24 h. (B) Comparing food intake in 4-h blocks. (C) Number of meals in 4-h blocks. (D) Time spent on feeding in 4-h blocks. (E) Food intake of fasted mice during 9:00−13:00. (F) Food intake of fasted mice during 16:00−20:00. (G) Quantitative RT-PCR revealed similar hypothalamic expression of genes involved in feeding control. 4-month-old female mice were assayed. Means±SEM for *n*≥5 animals per group are represented. *, ** and ***, *p*<0.05, *p*<0.01 and *p*<0.0001, respectively, by Bonferroni post-tests following ANOVA.

Food intake was further characterized in female mice prior to observable differences in body weight. The accumulative food intake during 24 h was decreased in mutant mice (Fig. 2A); moreover, this decrease was caused by significantly reduced food intake between 14:00 pm and 22:00 pm, which is the time that food consumption is highest in control mice (Fig. 2B). Between 22:00 pm and 14:00 pm, mutant mice had similar food intake compared to control mice. Reduction of food intake in mutant mice is not explained by the number of meals or time spent feeding, especially during 18:00 pm to 22:00 pm (Fig. 2C and D).

Fasting abolished the food intake difference between control and mutant mice, as fasted mutant mice had similar food intake compared to fasted control mice during 9:00 am and 13:00 pm (Fig. 2E), when non-fasted control and mutant mice showed no food intake difference, and during 14:00 pm to 18:00 pm (Fig. 2F), when non-fasted mutant mice had reduced food intake. These data suggest that the effects of fasting on food intake override the effects of *Immp*2*l* gene mutation. Neural peptide Y, agouti antigen-related peptide, pro-opiomelanocortin-derived peptides, melanin-concentrating hormone, and orexin all control appetite via neural interactions in the hypothalamus. We examined the expression of *Npy*, *Agrp*, *Pomc*, *Mch*, and *Orexin*, genes coding for these appetite controlling peptides, and found no difference between control and mutant mice (Fig. 2G).

### Pair-feeding equalizes body weight and adiposity of control and mutant mice

**Fig. 3:**
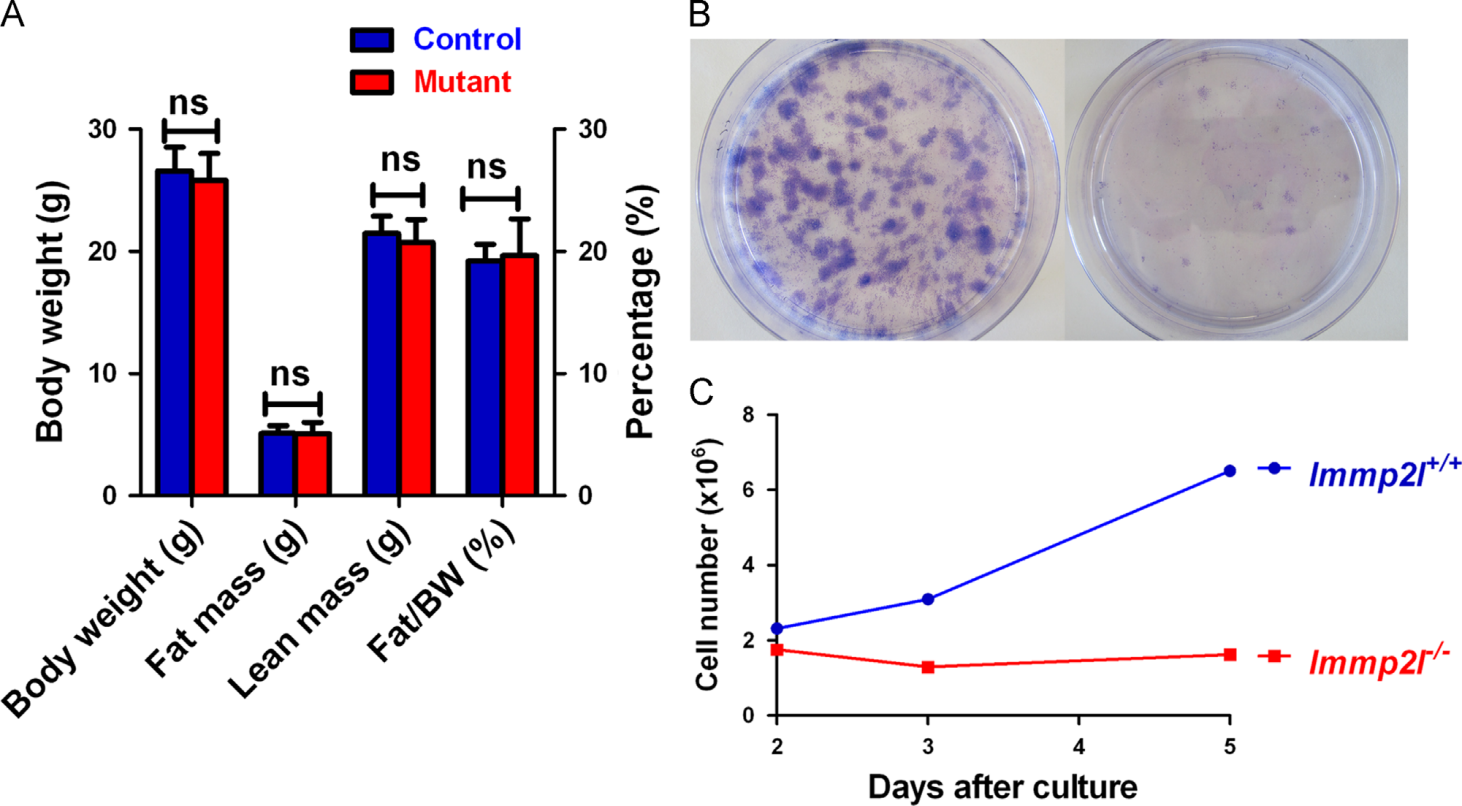
Pair-feeding equalized the body weight and fat composition of control and mutant mice. (A) Body weight and adiposity of mutant mice and normal mice fed the food consumed by mutant mice. MRI was conducted on female mice at the age of 6 months (*n*=5 for each group). (B) Fewer and smaller ADSC colonies were obtained from fat tissue of mutant mice. (C) Fewer cells could be obtained from mutant ADSC after in vitro culture. For (B) and (C), normal mice were pair-fed. Data represented three pairs of mice assayed at the age of 22−25 months.

To examine whether food intake accounts for the reduced adiposity of mutant mice, control mice were fed the equivalent amount of food consumed by mutant mice from the age of 6 weeks. Pair-fed control mice and ad libitum-fed mutant mice had similar body weight up to the age of 20 months (Fig. 1A, dashed line). In addition to similar body weight, they also had similar body compositions (Fig. 3A). These data demonstrate that reduced body weight of mutant mice is the result of reduced food intake.

We previously reported that adipose-derived stromal cells (ADSCs) isolated from older mutant mice showed impaired proliferation [38]. The observation of reduced food intake and fat accumulation in mutant mice prompted us to examine whether impaired ADSC proliferation of mutant mice is a consequence of reduced food intake and adiposity. Since pair-fed control mice have similar body weight and adiposity compared to mutant mice, we isolated ADSC from age-matched pair-fed control mice and ad libitum-fed mutant mice at ages 22–25 months and cultured these cells at low density to assess colony formation. Colonies obtained from control mice were significantly larger in number and size compared to colonies observed from the mutant mice (Fig. 3B). ADSC from control and mutant mice were both cultured for one week, and cell numbers were counted at time of cell passage. More cells were obtained from control mice than from mutant mice (Fig. 3C). These observations were similar to previous observations made from ad libitum-fed control and mutant mice [38].

### Mutant mice have normal energy expenditure

**Fig. 4:**
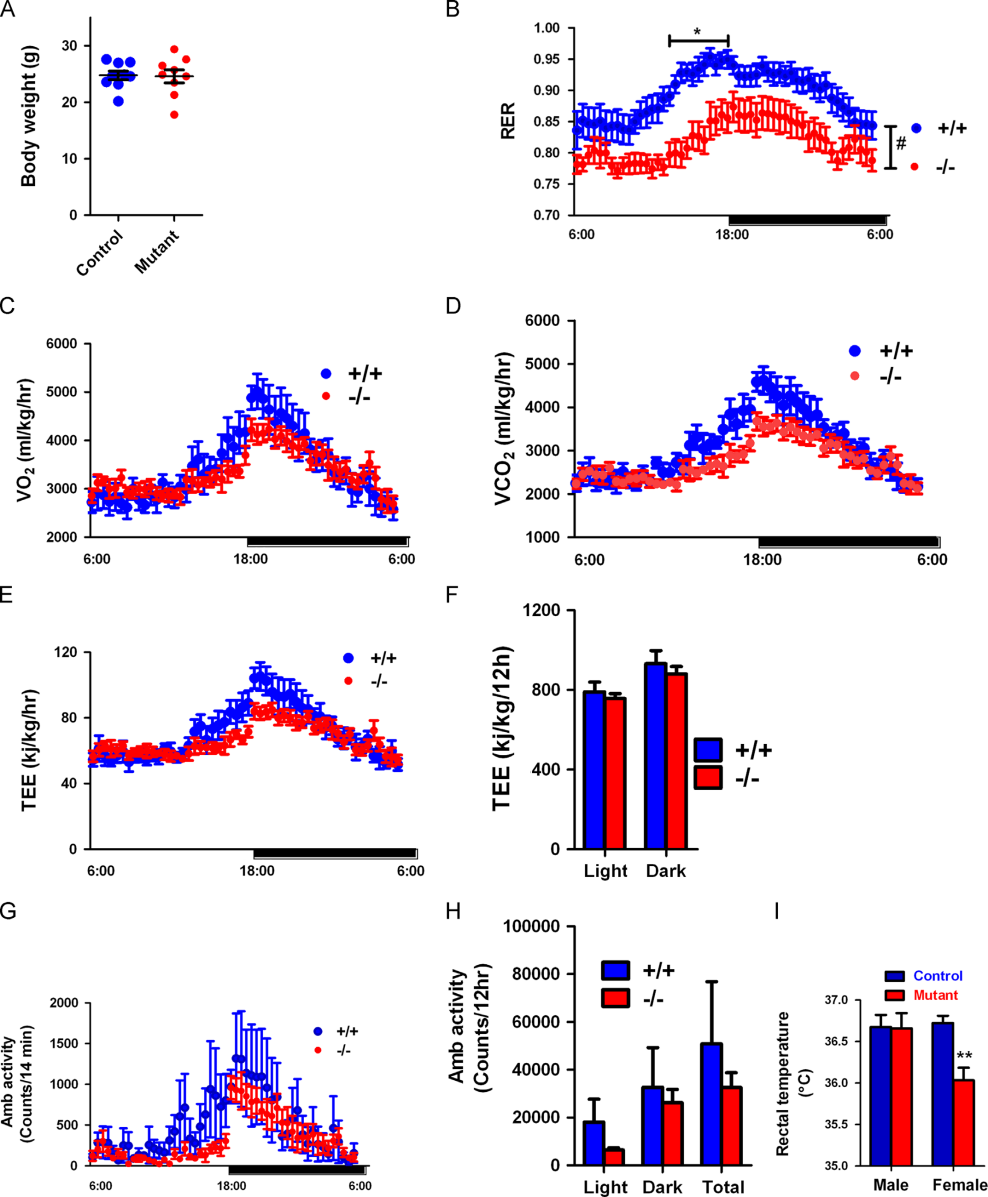
Normal energy expenditure of mutant mice. (A) Similar body weight of mice in metabolism analysis. (B) Mutant mice have reduced respiratory exchange ratio (RER). (C) Profile of oxygen consumption. (D) Profile of carbon dioxide production. (E) Profile of total energy expenditure. (F) Total energy expenditure during light and dark cycle. (G) Profile of ambulatory activity. (H) Total ambulatory activity during light and dark cycle. (I) Rectal temperature. Means±SEM for *n*≥5 animals per group are represented. *, ** and ***, *p*<0.05, *p*<0.01 and *p*<0.0001, respectively, by Bonferroni post-tests following ANOVA.

Energy expenditure was further examined by indirect calorimetry in 12 to 16-week-old control and mutant mice prior to observable differences in body weight (Fig. 4A). The respiratory exchange ratio (RER) of mutant mice was significantly lower than that of control mice, suggesting a preference of mutant mice to metabolize fat as an energy source (Fig. 4B). This difference in RER is unlikely to be a feedback response to variable fat composition as there is no difference in body weight, or fat composition, at this age. While the cause of reduction in RER in mutant mice is not clear, this may be an alternate mechanism by which mutant mice achieve reduced adiposity after 5 months. Oxygen consumption (Fig. 4C), carbon dioxide production (Fig. 4D), total energy expenditure (Fig. 4E and F), and locomotor activity (Fig. 4G and H) were all similar between control and mutant mice at age 3–4 months. Rectal temperature was not statistically different between male control and mutant mice, but was significantly reduced in female mutant mice (*p*<0.01) (Fig. 4I). The cause of decreased body temperature in mutant female mice is unclear.

### Mutant mice had reduced hypothalamic cGMP concentration

**Fig. 5:**
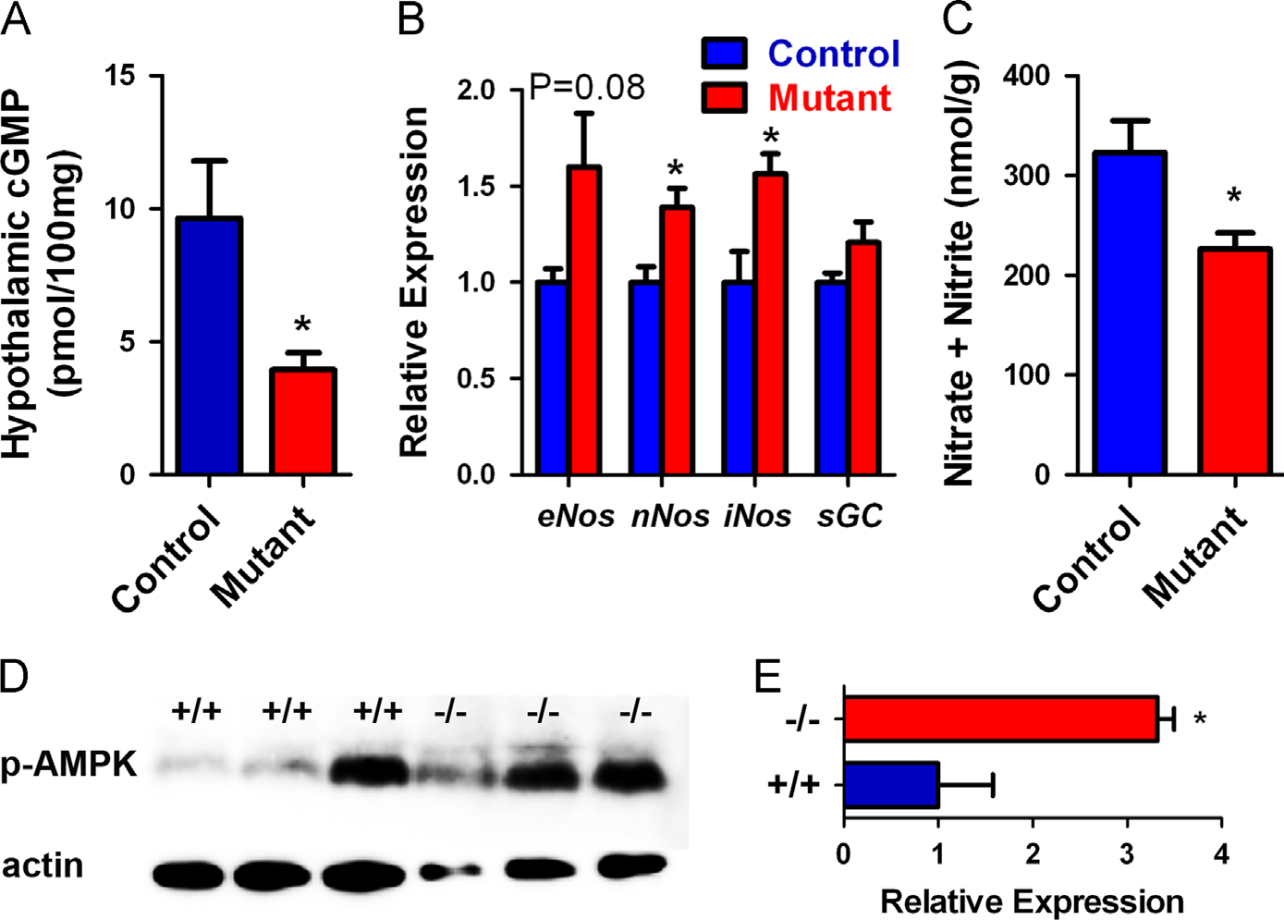
Analysis of NO signaling and AMPK phosphorylation. (A) Mutant mice have decreased hypothalamic cGMP concentration. (B) Quantitative RT-PCR analysis of hypothalamic expression of genes encoding nitric oxide synthase (NOS) and soluble guanylate cyclase (sGC). (C) Mutant mice had decreased free hypothalamic nitrate and nitrite content. Means±SEM for *n*≥5 animals per group are represented. (D) Western blotting analysis of AMPK phosphorylation. (E) Relative expression of phosphorylated AMPK by integrated density of ImageJ software. For (A)−(C) and (E), * and **, *p*<0.05 and *p*<0.01, respectively, by *t*-tests.

As an important signaling molecule, NO mediates feeding; furthermore, our previous data indicate that mutant mice had elevated mitochondrial superoxide generation, which caused erectile dysfunction and defective oogenesis, which may result from reduced NO bioavailability [35]. Therefore, our study focused on the relationship between reduced food intake and NO bioavailability in the hypothalamus. In the central nervous system, soluble guanylate cyclase is the primary receptor for NO, which transforms guanosine monophosphate (GMP) into cyclic GMP (cGMP). Due to the difficulty in assaying NO, we measured the concentrations of cGMP, the product of NO action, in the hypothalami of control and mutant mice. Mutant mice, indeed, had significantly reduced hypothalamic cGMP concentration (*p*<0.01, Fig. 5A), indicating low NO in the hypothalamus of mutant mice. Expression of soluble guanylate cyclase, the major receptor for NO, and nitric oxide synthases, which are the genes that synthesize NO in vivo*,* was not reduced in mutant hypothalami (Fig. 5B). On the contrary, mutant mice had significantly increased expression of *nNOS* and *iNOS* (*p*<0.05). The increased expression of *nNOS* and *iNOS* are consistent with a feedback response to reduction in NO bioavailability in the mutants.

NO has three principal reactions in vivo: (1) binding to guanylate cyclase to stimulate cGMP production, (2) reacting with oxyhemoglobin to form nitrate, and (3) reacting with superoxide to form peroxynitrite. Modification of macromolecules by peroxynitrite limits the availability of NO to form nitrate. Since mutant mice generate higher levels of superoxide to react with NO, more peroxynitrite is produced and less NO will be converted to free nitrate. As expected, hypothalamic lysates from mutant mice had significantly reduced nitrate/nitrite content than those from control mice (*p*<0.05) (Fig. 5C). These data support our hypothesis that higher mitochondrial superoxide generation in the mutant mice will react with NO to form peroxynitrite and less NO is available for activating its receptor guanylate cyclase.

We examined the phosphorylation of AMPKα in the hypothalamus of mutant mice and found that it was hyperphosphorylated compared to the control mice (Fig. 5D and E). Thus, decreased food intake observed in mutant mice is not related to insufficient AMPK phosphorylation, but may be related to downstream targets of AMPK.

### Immp2l mutation has little effects on the food intake or obesity of ob/ob mice

**Fig. 6:**
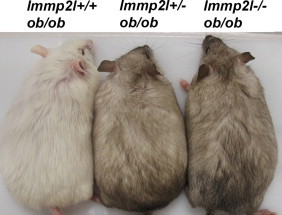
*Immp*2*l* mutation in *ob/ob* mice did not significantly affect their obesity. Picture was taken on male mice at the age of 9 weeks.

Nitric oxide is produced and functions both pre- and post-synaptically [44]. Recently, it has been shown that knockout of leptin receptor in *Nos*1-positive neurons produces obesity similar to ob/ob mice [45]. Therefore, we studied the effects of *Immp*2*l* mutation on food intake of *ob/ob* mice to determine whether *Immp*2*l* mutation could affect food intake through perturbation of leptin signaling. We introduced the *Immp*2*l* mutation to *ob/ob* mice, then we assessed at the age of 8 weeks that *Immp*2*l*^*−/−*^*;ob/ob* mice had similar food intake compared to *ob/ob* mice (8.2±0.28, *N*=10 for *ob/ob* mice; 7.7±0.34, *N*=4 for *Immp*2*l*^*−/−*^*;ob/ob* mice, *p*=0.35, data were from equal numbers of male and female mice, which were not significantly different). In addition, obesity of *Immp*2*l*^*−/−*^*ob/ob* mice was comparable to obesity in *ob/ob* mice (Fig. 6). Our data indicate that *Immp*2*l* mutation has little effect on the food intake and obesity of *ob/ob* mice.

## Discussion

In the present study, we report that *Immp*2*l* mutant mice have reduced body weight and adiposity after the age of 5 months, compared with control mice. Our data show that decreased body weight and adiposity is the direct result of food intake reduction. First, food intake is reduced by 30% in mutant mice; moreover, the degree of reduction did not affect the development of mutant mice, but it did reduce energy surplus as the mice reached adulthood, and they did not gain weight. Second, feeding control mice the equivalent amount of food consumed by mutant mice equalized body weight and adiposity of the control mice. Third, energy expenditure of mutant mice was not significantly affected. Mutant mice do exhibit higher RER than control mice, which suggests a preference for lipids as the substrate. Thus, this may be one of the mechanisms leading to reduced adiposity. Further studies will be needed to confirm the mechanism of RER change in the mutant mice.

As previously reported by our group, mitochondria from mutant mice generated increased levels of superoxide, which reacts with NO and reduces the bioavailability of nitric oxide and may explain the erectile dysfunction observed in mutant males [35]. Nitric oxide has been shown to stimulate feeding in rodents in multiple reports [6–13]. Reduced bioavailability of nitric oxide in the brain of mutant mice may also be the mechanism for food intake decrease. To support this view, we observed that hypothalami of mutant mice have reduced levels of cGMP, the product of nitric oxide signaling [44]. Although mutant mice had reduced brain cGMP levels, expression of nitric oxide synthases, genes encoding the enzymes that synthesize nitric oxide, was increased. This is consistent with reduced nitric oxide bioavailability due to excessive mitochondrial superoxide negation in the mutants. We injected control and mutant mice with NO donor nitroprusside, and found that food intakes in control and mutant mice were both increased but showed no difference between genotypes (data not shown). This suggests that the NO signaling machinery in mutant mice is functional, and that the exogenous provided NO is able to saturate the sCG for maximal NO signaling after reaction with mitochondrial superoxide.

A direct comparison of NO concentrations between control and mutant mice could strengthen our argument. However, measurement of NO concentration in biological samples is a difficult task. Fluorescent probes usually lack the specificity, and electrochemical sensors are in developing stages and not easily accessible. Restoring food intake of mutant mice with antioxidants could be another way to prove our hypothesis. These novel antioxidants must be blood-brain barrier permeable, can either reduce mitochondrial superoxide generation, or dismutate superoxide at a speed faster than the reaction between superoxide and NO. To the best of our knowledge, antioxidants meeting these criteria are unavailable so far.

We observed that, AMPK, whose phosphorylation stimulates NO production and food intake [27,28], is hyperphosphorylated in mutant mice. This might be a feedback response to insufficient NO signaling. Peroxynitrite has been shown to activate AMPK [46]; and, hyperphosphorylaion of AMPK in the mutants may be the result of increased production of peroxynitrite in the mutants.

Currently, the neural circuits affected in the mutants are unclear. It has been shown recently that knockout of leptin receptor in *Nos*1-positive neurons produces obesity similar to *ob/ob* mice [45], which indicates that these neurons are the major sites of leptin signaling. The role of NO in leptin signaling remains unclear. We mutated *Immp*2*l* gene in *ob/ob* mice and found that it had little effect on the food intake and obesity of *ob/ob* mice. Among the possible explanations for our observations are (1) *Immp*2*l* mutation has no effect on NO signaling, and *Immp*2*l* mutation in *ob/ob* mice would have little effect on leptin signaling; and (2) *Immp*2*l* mutation will affect NO signaling, but NO signaling does not play a critical role in leptin signaling. We do not believe the first explanation is a possibility since we observed erectile dysfunction and bladder dysfunction in mutants, which suggests abnormal NO signaling [35,36]. The second explanation is more likely, and would suggest that alternate pathways that regulating feeding are affected in *Immp*2*l* mutant mice, and the effects of these pathways are overridden in the absence of leptin. Supporting this view, *Immp*2*l* mutant mice ate as much as control mice after fasting, and leptin level was decreased [47].

Adipose-derived stromal cells (ADSCs) isolated from older mutant mice did not proliferate well [38]. Since ADSC from mutant mice still showed impaired proliferation compared with those from pair-fed control mice, the defects may not be secondary to changes in adiposity. Furthermore, age-related defects such as ataxia in *Immp*2*l* mutant mice may not be secondary to reduction in food intake either. We observed that pair-fed control mice do not exhibit ataxia at the age of 16 months as the ad libitum-mutant mice do at the same age. A 30% diet restriction has been shown to slow aging [48,49]. Despite the fact that *Immp*2*l* mutant mice have reduced food intake, early onset of age-associated defects has been observed [38], which highlights the important role of ROS in aging.

In conclusion, *Immp*2*l* mutant mice have reduced body weight and adiposity resulting from reduced food intake, which in turn, could be linked to insufficient NO signaling, due to negation of NO by superoxide.

## Figures and Tables

**Table 1:** Blood parameters of control and mutant mice^a^.

**Measurement**	**Age (m)**	**Control**	**Mutant**	***p***
**Letpin (ng/ml)**	3	2.6±0.5 (8)	4.0±1.0 (6)	ns
	16	20.4±3.0 (5)	10.7±1.2 (8)	**0.03**
**Insulin (ng/ml)**	3	0.33±0.01 (11)	0.33±0.02 (10)	ns
	16	1.18±0.30 (18)	0.44±0.04 (18)	**0.028**
**FFA (μM)**	3	514.1±30.4 (10)	580.4±38.8 (10)	ns
	16	1210±80.7 (9)	1278±94.6 (9)	ns
**Cholesterol (mg/dl)**	16	219.3±11.0 (10)	224.7±10.3 (9)	ns
**Triglyceride (mg/dl)**	12	124.2±9.6 (18)	53.3±8.5 (7)	**0.0003**

a Mean±SEM are present with number of female mice assayed in parentheses. *p*<0.05 by *t*-test was taken as significant. ns, Not significant.

**Table 2:** Food intake of control and mutant mice^a^.

**Mice**		**Controls**	**Mutants**	***p***
Female (2-3 m)	Body weight (g)	23.3±1.1 (12)	23.4±0.8 (17)	ns
	Food/mouse/day (g)	4.2±0.14 (5)	3.0±0.08 (5)	0.00005
	Daily food/bodyweight	0.172±0.003 (5)	0.123±0.007 (5)	0.0003
Male (13 m)	Body weight (g)	44.2±1.6 (8)	33±1.7 (8)	0.00036
	Food/mouse/day (g)	4.3±0.20 (8)	4.0±0.10 (8)	0.17

a Mean±SEM for number of animals in parentheses are present. *p*<0.05 considered significant. ns, Not significant.
